# Parallel Acquisition of Uncorrelated Sequences does Not Provide Firm Evidence for a Modular Sequence-Learning System

**DOI:** 10.5334/joc.258

**Published:** 2023-01-18

**Authors:** Marius Barth, Christoph Stahl, Hilde Haider

**Affiliations:** 1 Department of Psychology, University of Cologne, DE

**Keywords:** implicit learning, single-system and dual-systems models, encapsulated processing modules

## Abstract

Dual-systems theories of sequence learning assume that sequence learning may proceed within a unidimensional learning system that is immune to cross-dimensional interference because information is processed and represented in dimension-specific, encapsulated modules. Important evidence for such modularity comes from studies investigating the absence or presence of interference between multiple uncorrelated sequences (e.g., a sequence of color stimuli and a sequence of motor keypresses). Here we question the premise that the parallel acquisition of uncorrelated sequences provides convincing evidence for a modularized learning system. In contrast, we demonstrate that parallel acquisition of multiple uncorrelated sequences is well predicted by a computational model that assumes a single learning system with joint representations of stimulus and response features.

The distinction between implicit and explicit learning is fundamental to theories of human learning. *Implicit* learning refers to learning that proceeds in the absence of awareness; *explicit* learning occurs when propositions are consciously formed about what is learned (Shanks, 2010). Important evidence supporting the distinction between implicit and explicit learning comes from work with the serial response time task (SRTT, Nissen & Bullemer, 1987). In this task, subjects are instructed to respond to sequentially presented stimuli with a corresponding response; typically, the stimuli are symbols at different screen locations, and the responses to be made are keystrokes on a computer keyboard. If a stimulus appears at one of the possible locations, the subject is to respond with the corresponding key. The locations of the stimuli are not randomly selected, but follow an underlying sequence (e.g. 3–4–2–1–3–1–2–4). Subjects show accelerated responses and/or lower error rates in the course of task processing; these performance gains are greater for transitions that follow the sequence than for transitions in which the sequence is violated. After completing the SRTT, subjects’ sequence knowledge is measured by some measure of explicit sequence knowledge. Importantly, subjects usually show no signs of explicit sequence knowledge. This simple dissociation between performance gains in the SRTT and subsequently recorded explicit sequence knowledge is considered to be one of the major pieces of evidence for the existence of implicit (in addition to explicit) learning processes.

There is no consensus on whether these two observed forms of learning are also based on two different learning mechanisms or systems, or whether both phenomena may be explained with one general learning mechanism. Cleeremans and Jiménez (2002) assume that implicit and explicit sequence learning are based on a single learning system. Rather, they explain the dissociation between performance effects in the SRTT and measures of explicit sequence knowledge by the fact that the measure of explicit sequence knowledge is less sensitive or reliable (Shanks & St. John, 1994; Timmermans & Cleeremans, 2015).

The assumption of a unitary system is opposed by the assumption of two systems. Keele, Ivry, Mayr, Hazeltine, and Heuer (2003) distinguish a unidimensional and a multidimensional learning system: In the multidimensional system, information of multiple *feature dimensions* can be processed together; the system is dependent on attention to protect it from overload. Processing in this system initially takes place automatically and without awareness, but the information processed in the system is in principle accessible to consciousness. In the unidimensional system, by contrast, processing takes place in a series of *encapsulated, dimension-specific modules*. The system operates independently of attention – it is only protected from overload because encapsulation and, hence, separate processing of stimulus and response features already protects the system from overload. Attributable to the strong separation of information, knowledge in this system is not accessible to consciousness. The two systems thus differ in terms of their dependence on attention, their accessibility to consciousness, and how strongly separated from each other features (from different dimensions) are processed.

What constitutes a *dimension* is, however, not firmly defined by the model; Keele et al. (2003) acknowledge that in the context of the SRTT, dimension has been used interchangeably with *modality*, but that distinctions within the motor system (e.g., hands vs. feet) as well as the visual domain (e.g., colors vs. shapes) might constitute separate dimensions. Goschke and Bolte (2012), by contrast, propose that the processing systems are separable by distinct functions, such as spatial attention, object recognition, phonological processing, and manual response selection. Recently, Haider and colleagues (Eberhardt, Esser, & Haider, 2017; Haider, Esser, & Eberhardt, 2018, 2020) proposed that the modules of the unidimensional learning system are separated along *abstract* feature dimensions such as shapes, colors, or spatial locations, not modality.

In addition to the dissociation of implicit and explicit knowledge, previous studies investigated the influence of attention (e.g., Rowland & Shanks, 2006) and the postulated encapsulation within the unidimensional system. As Keele et al. (2003) point out, important evidence for encapsulated processing comes from studies investigating interference between (1) learning of a sequence and a secondary task and (2) multiple sequences that may be learned in parallel. In the following, we focus on the parallel acquisition of multiple sequences, but the line of argument similarly applies to studies using a secondary task.

Studies investigating such parallel acquisition repeatedly found evidence that parallel acquisition is indeed possible: In an early study by Mayr (1996), participants were instructed to discriminate between objects that were presented in different locations, where objects and locations followed two independent sequences. Mayr (1996) found learning of both sequences even for participants who were not aware of the sequences, and concluded that implicit learning “may be supported by several, distinct modules that are linked to different attentional subsystems and thus allow parallel acquisition of multiple, independent regularities”. More recently, Goschke and Bolte (2012) found parallel acquisition of uncorrelated spatial-visual, spatial-motor, phonological and color sequences in a series of three experiments. Moreover, they also tested the effects of a secondary distractor task and found that a spatial distractor task selectively disrupted the acquisition of a spatial sequence, while adding a phonological distractor task selectively disrupted learning of a letter sequence. They interpreted both findings as evidence for modularization within a unidimensional learning system.^1^ Eberhardt et al. (2017) and Haider et al. (2018) also found that multiple sequences can be learned in parallel; however, if the sequences overlap in an abstract feature dimension (for example, a visuo-spatial stimulus sequence and a motor-spatial response sequence), they cannot be learned in parallel. The authors concluded that interference occurs within a module that processes spatial features – encapsulation within the unidimensional learning system would be best described in terms of abstract feature dimensions.

To summarize, these studies demonstrate that, if boundary conditions are met, participants are well able to learn multiple sequences in parallel. *Do these findings provide evidence for modularized processing, though?*

The common rationale underlying the above studies is that parallel acquisition is possible only if the processing of both sequences occurs in a modularized fashion; if, instead, sequences were processed jointly, they could not be learned because *destructive interference* between sequences would eliminate learning. Accordingly, the above-presented studies tested whether above-zero learning can be found for both sequences.

However, finding above-zero learning is not sufficient to conclude that no interference between sequences occurred. Instead, it is conceivable that the observed pattern of results may be the result of *incomplete destructive interference*, where learning of each sequence is hampered, but still strong enough to yield above-zero learning scores. Yet another alternative explanation might be *constructive* interference, where the parallel processing of one sequence may foster processing of the other, possibly by redundancies between features of sequences. We deem both alternative explanations well compatible with joint (i.e., non-encapsulated) processing of multiple sequences. Hence, finding above-zero learning scores for multiple sequences cannot be interpreted as evidence for a modularized sequence learning system.

By contrast, if it was possible to demonstrate *absence of interference* between multiple sequences, such a finding would provide much more compelling evidence in favor of modularized processing. Mayr (1996) already acknowledged the problem and therefore, in his Experiment 2, compared learning of both sequences in a dual-sequence condition (where both sequences were present) with learning under conditions where only one of the two possible sequences was present. He found that learning of the individual sequences was *superior in the dual-sequence condition* compared to the single-sequence conditions. In our view, such a finding is inconsistent with absence of interference and may be better explained with (1) an increase in attentional resources devoted to the task under dual-sequence conditions *or* (2) constructive interference between sequence representations. Such constructive-interference (i.e., super-additive learning) effects could possibly be explained with memory theories that assume that associative binding in the medial temporal lobe is hierarchically organized (Shimamura & Wickens, 2009), where the parallel activation of multiple lower-level units results in the additional activation of higher-order units: Such higher-order activations in turn disproportionately increase the probability of reinstating lower-level units, yielding superadditive learning effects.

## Aims of the present study

The present study is aimed at demonstrating that a computational model that assumes joint processing of stimulus and response features can predict the parallel acquisition of multiple uncorrelated sequences. Put differently, we aim to demonstrate that finding *incomplete* destructive interference between multiple sequences is not sufficient to preclude a single-system explanation of such parallel learning.

For this purpose, we applied the memory model that Jamieson and Mewhort (2009) developed for a standard SRTT (with a single motor sequence) to a study design with two uncorrelated sequences: It is assumed that features of the stimuli and response of each trial, together with the same information from the preceding trial, are represented as another episode (another *instance*) in a single memory storage. In each trial, the current stimulus, in addition to stimulus and response of the preceding trial, serves as a *probe*; the similarity of this probe to all instances in the memory store determines the intensity and content of the memory *echo*, which then determines which response is executed.

## Method

We simulated 1,000 data sets each comprising 40 participants who worked on six blocks (180 trials per block) of an SRTT procedure where stimuli followed a seven-item probabilistic sequence with 50% regular transitions (no direct repetitions). Responses followed a six-item probabilistic sequence with 50% regular transitions (no direct repetitions); both sequences were generated independently from each other. Such experiments are typically implemented by presenting color stimuli and switching the color-to-response-key mapping in a trial-wise fashion.

In such a design, parallel acquisition of both sequences can be tested by comparing response times (and error rates) for regular vs. non-regular stimuli and of regular vs. non-regular responses: Over learning blocks, responses should become fastest (error rates should become lowest) for trials with stimuli that follow the stimulus sequence, and also require a response that follows the response sequence. By contrast, responses should become slowest (error rates should become highest) for trials that violate both sequences (i.e., a non-regular stimulus presented, and the required response also violates the sequence of responses). For trials with regular stimuli but non-regular responses, or trials with non-regular stimuli but regular responses, performance should be in-between these two extremes.

### Model specification

The Jamieson and Mewhort (2009) model is an application of Hintzman (1984)’s Minerva 2 model: Memory is represented as a matrix where each row (vector) consists of the information that is encoded in a single episode. For each episode, or instance, a new row is added at the bottom of the memory matrix.

To simulate the SRTT, we first constructed 13 vectors, seven to represent stimulus features (e.g., colors), and six to represent features of the motor responses. Each vector was of length 30 with features *x_j_* randomly drawn with *P*(*x_j_* = +1) = *P*(*x_j_* = –1) = .5. Vectors also fulfilled the additional constraint that pairwise similarities between two vectors (as measured by their vector cosine) were not larger than .4. Feature vectors remained constant within each simulation.

For each trial *i* of the experiment, a new instance was added at the bottom of the memory matrix, containing the vectors representing the current stimulus **S***_i_*, the previous stimulus **S**_*i*–1_, the response given **R**_*i*_, and the response given on the previous trial **R**_*i*–1_. We initialized memory with 200 pre-experimental traces where stimuli and responses were randomly chosen with equal probability (i.e., no sequential structure was present). For these initial memory traces and the study phase, we assumed a *learning rate L* = .8: Features were stored with a probability of .8 in memory. If a feature is not stored, its position in the memory vector is replaced with a zero.

Memory is probed with the subset of information that is already available before a response is selected: The probe vector **P** is constructed by concatenating the vectors representing the currently-presented stimulus **S**_*i*_, the stimulus of the preceding trial **S**_*i*–1_, and the response of the preceding trial **R**_*i*–1_. For each instance *i* already stored in memory (i.e., for each row of the memory matrix *M*), its similarity *S_i_* with the probe vector is given by the vector cosine of the probe and the corresponding features *j* = 1, …, *J* in memory,

{S_i} = \frac{1}{J}\sum\limits_{j = 1}^J {{P_j}} \cdot {M_{ij}}

The *activation* of each instance *i* is then calculated as the cube of its similarity

{A_i} = S_i^3

Memory information is returned in the *echo*. The echo’s *content* **C** = {*C*_1_, …,*C_J_*}) is the weighted sum of the traces stored in memory. For the *j*th feature, it is given by

{C_j} = \sum\limits_{i = 1}^I {{A_i}} \cdot {M_{ij}}

The echo’s *intensity* is the sum of the products of probe features and echo content.

I = \sum\limits_{j = 1}^J {{P_j}} \cdot {C_j}

Jamieson and Mewhort (2009) used an iterative-resonance model to simulate response times from Minerva’s cued-recall mechanism. We replaced this mechanism because it makes unrealistic predictions for the SRTT: In an SRTT with deterministic sequences, it predicts perfect performance (i.e., no response errors); for an SRTT with probabilistic sequences, it predicts no errors for regular stimuli and only errors for non-regular stimuli. We, therefore, replaced the iterative-resonance part of the model with a drift-diffusion model that predicts more-plausible patterns of response times and error rates. The diffusion model (Ratcliff, 1978; Wagenmakers, 2009) has been developed to model response times and error rates in two-alternative speeded choice tasks. It assumes that on a given trial, participants sequentially sample information in a noisy fashion. The decision process starts at an initial value determined by the decision bias *β*. Evidence is accumulated with average rate *δ* (the drift rate); if the evidence for one alternative is strong enough (i.e., if one of the two decision thresholds is reached), a response is selected. Extradecisional components of the response time (e.g., stimulus encoding and response execution) are captured by non-decision time *τ*.

To model more than two response alternatives, it is common practice to use *accuracy coding*, where upper-threshold responses correspond to correct responses and lower-threshold responses correspond to errors (Voss, Nagler, & Lerche, 2013). To model the SRTT, we calculated, for each of the *k* = 1, …, *K* possible responses, the respective similarity between its vector representation *R_k_* and the current-response part of the echo content C* (i.e., the response-echo similarities) as the vector cosine plus 1, yielding an unsigned measure of similarity:

1 + \cos ({\bf C}^{*},{{\bf R}_k}) = 1 + \frac{{{\bf C}^{*} \cdot {{\bf R}_k}}} {\Vert{{\bf C}^{*}\Vert\ \Vert{{\bf R}_k}}\Vert}

The response-echo similarity for the correct response is then divided by the sum of response-echo similarities of all possible responses *R_k_*, k = 1, …*K*, yielding the *signal-to-noise ratio* SNR:

{\rm{SNR}} = \frac{{1 + \cos ({\bf C}^{*}, {{\bf R}_m})}}{{\sum\nolimits_{k = 1}^K 1 + \cos ({\bf C}^{*}, {{\bf R}_k})}}

The product of echo intensity *I* and signal-to-noise ratio SNR is fed into the drift-rate parameter *δ* of the drift-diffusion model. The other parameters of this standard diffusion model (boundary separation *α*, relative starting point *β*, and non-decision time *τ* were set to constant values. Response times and error rates are then described by the Wiener distribution *W* (e.g., Navarro & Fuss, 2009).

(RT,R)\sim {\cal W}(\alpha = 1.5,\beta = .2,\delta = {\rm{max}}(3,{\rm{SNR}} \times I),\tau = .3)

If the drift-diffusion process terminated in the upper threshold, this was coded as a correct response (i.e., the response vector that belonged to the to-be-pressed response). If, instead, it hit the lower threshold, this was coded as an erroneous response and one of the five vectors that corresponded to incorrect responses was randomly chosen with equal probability.

After the response was given, a new instance was added at the bottom of the memory matrix, containing the vectors representing the current stimulus **S***_i_*, the previous stimulus **S**_*i*–1_, the response given **R**_*i*_, and the response given on the previous trial **R**_*i*–1_. All features were stored with learning rate *L* = .8; if a feature was not stored in memory, its position in the memory vector was replaced with zero.

## Results

We used R (Version 4.2.1; R Core Team, 2022) and the R packages *papaja* (Version 0.1.1; Aust & Barth, 2022), *rtdists* (Version 0.11.5; Singmann, Brown, Gretton, & Heathcote, 2022), and *tinylabels* (Version 0.2.3; Barth, 2022) for all simulations and analyses. All code and materials necessary to reproduce the manuscript and results are available from https://github.com/methexp/cpl-minerva.

Learning of (probabilistic) sequences in an SRTT is typically tested by comparing response times and error rates for regular (i.e., sequenced) and non-regular trials across blocks. In an extended design with two independent sequences, we can distinguish four different trial types: (1) Both stimulus and response adhere to their respective sequences, (2) only the stimulus is regular, but a non-regular response is required, (3) only the response is regular, but a non-regular stimulus is presented, and (4) both stimulus and response do not follow their respective regularities. If both sequences are acquired, it should be possible to observe a performance advantage for trials that follow both sequences compared to trials that follow only one of the two sequences; moreover, trials that follow only one of the two sequences should still result in better performance compared to fully non-regular trials. By contrast, if it is not possible to learn both sequences in parallel, performance for all types of trials should be comparable (i.e., there should be no differences in performance between trial types).

**Figure 1:**
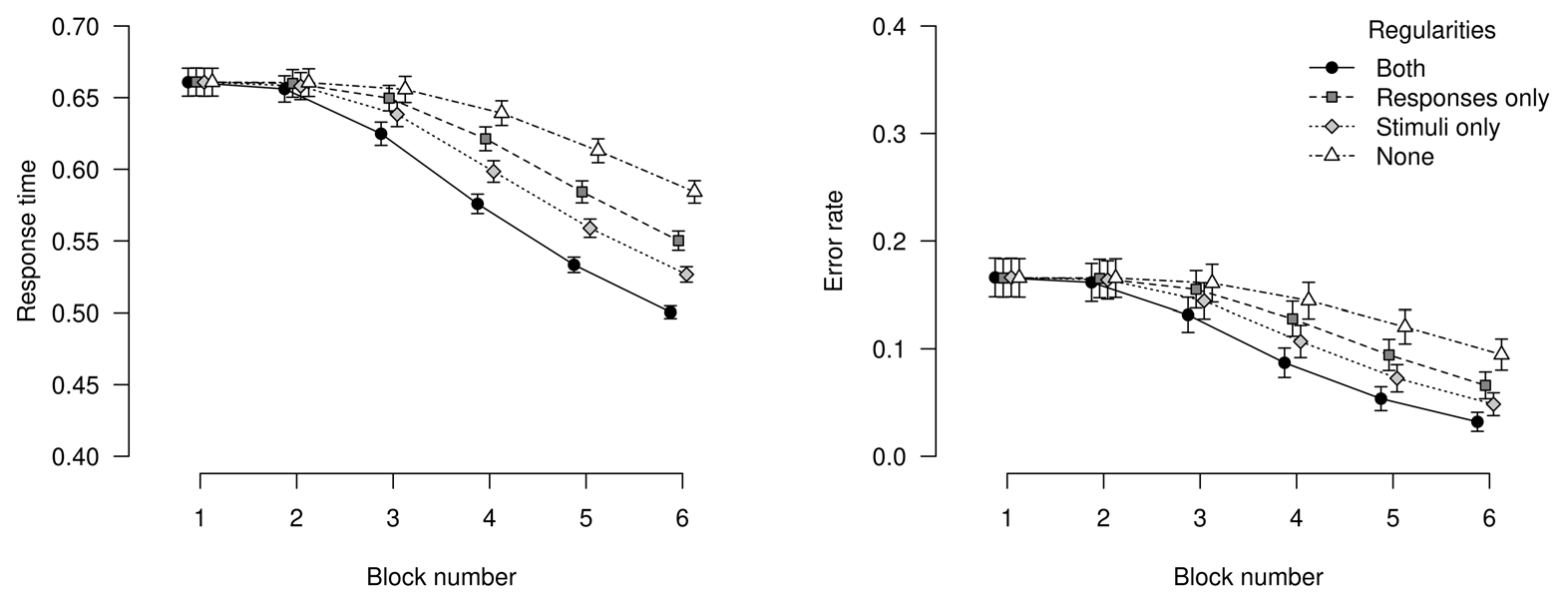
Response times and error rates from simulations. Left panel: Mean response times for non-error trials, averaged over simulations. Right panel: Proportion of erroneous responses, averaged over simulations. Error bars represent the average width of 95% within-subjects confidence intervals.

Figure 1 shows response times and error rates as predicted from our simulation model. Response times decrease over learning blocks: This performance gain is most pronounced for trials where both stimulus (color) and response (location) adhere to their respective sequence. For trials that violate both stimulus and response sequences, responses are slowest; for trials that follow only one of the two sequences, performance is in-between fully regular and fully non-regular trials. This pattern of results is mirrored in error rates: Error rates decrease most for trials following both sequences and decrease least for trials that do not follow either sequence. Again, trials that follow only one of the two sequences indicate in-between performance. This pattern of results indicates that both sequences were learned by the model.

## Discussion

A core tenet of the dual-systems view (Keele et al., 2003) is that sequence learning may proceed in a unidimensional learning system where information is processed in encapsulated modules, with each module processing only a single dimension of information (i.e., a single feature dimension). As a consequence of such strong modularization, learning in this system is assumed to be immune to cross-dimensional interference.

The parallel acquisition of multiple uncorrelated sequences has been considered to provide crucial evidence for such encapsulated processing in an implicit learning system. However, such reasoning rests on the critical assumption that the parallel acquisition of sequences is not possible in a unitary learning system because destructive interference would eliminate learning. Here, we applied an instance model of sequence learning that assumes a unitary knowledge base of information and tested whether such a model can predict the parallel acquisition of multiple uncorrelated sequences. We found that this is indeed the case, indicating that such parallel acquisition alone cannot provide decisive evidence for encapsulated learning in dimension-specific modules.

### Limitations and open questions

An important question that arises from our simulations is whether the MINERVA model used is indeed incompatible with the view that stimulus and response features are processed in separate, functionally independent modules. One might argue that, in MINERVA, stimuli and responses are represented by distinct sets of fixed features, resulting in separate representations of stimuli and responses, and these could in turn be processed in separate systems (e.g. processing modules). In other words, independence may be unfairly built into the model by a specific choice of implementation; and the simulations’ success in accounting for the dissociation would thus not be an argument against but instead an example of independent modules. There are several reasons for why this is not the case.

First, the fixed manner in which MINERVA assigns features to vector positions (i.e., a given feature is always stored in a specific position of the vector; the value in that position indicates whether the feature is present or absent) is not an arbitrary choice; it is essential for the model’s ability to store information. Fixed features are necessary for a memory model such as MINERVA (which was originally developed as a theory of episodic memory) to encode and retrieve information because vector positions are used to compute similarities across instances; if feature representations changed or fluctuated across trials, it would be impossible to retrieve meaningful information from memory. This issue is, however, unrelated to the question of whether features are processed in separate modules or a unitary system: A memory model with separate storage modules (for instance, a version of MINERVA with two distinct memory matrices) could also only learn with such a fixed assignment.

Second, the model’s success does not depend somehow on the operation of distinct processes. It applies a single set of processes to its single memory matrix; it does not apply separate or independent processes to different subsets or sub-modules. It does not (and could not, without modifications) distinguish between specific feature subsets except where required by the task (e.g., is a feature part of the probe or not?).

Third, and most importantly, whereas stimuli and responses are represented by separate sets of features, these are not functionally independent; to the contrary, they are associated because they are stored in the same instance. To illustrate, it is helpful to note that MINERVA is mathematically equivalent to an auto-associative tensor memory (Kelly, Mewhort, & West, 2017): Storing features into instances is equivalent to increasing the associative weights between feature nodes, irrespective of whether they represent stimulus or response features, and what is returned by memory is determined by the associative weights between features. In other words, by adding another instance to memory, the change in associative strength *within* feature dimensions is governed by the exact same computational rule as the change in associative strength *between* feature dimensions. In a study design as discussed here, with uncorrelated sequences (of stimuli and responses), the associative strengths between stimulus and response features will indeed remain uninformative because the feature dimensions are uncorrelated. However, this is a feature of the learning environment, not the model. The model makes strong and empirically testable predictions for correlated features that are incompatible with modularization: For instance, it predicts that sequences that span across dimensions (e.g., sequences where only the combination of available information from both feature dimensions yields a performance advantage) can be learned. To the best of our knowledge, this prediction has only been tested once in the sequence learning literature (suggesting multimodal sequence learning is indeed possible, Kemény & Meier, 2016). It also predicts that correlated sequences should be better learned than uncorrelated sequences, a pattern that could explain why Meier and colleagues (Meier & Cock, 2010; Meier, Weiermann, & Cock, 2012) found learning only for correlated, but not uncorrelated, streams of information.^2^

It is important to note that MINERVA is not committed to a specific neural representation or specific brain structures involved. Whereas obviously compatible with a single unitary processing system, it describes a general memory algorithm that may also be implemented independently in several different subsystems – or even in a (partially) modularized manner, as long as the modules are interconnected in a meaningful way to accommodate the functional predictions described above. In the statistical-learning literature, Frost, Armstrong, Siegelman, and Christiansen (2015) argued for domain-specific processing units that implement general learning principles and feed into multimodal or partially shared neural networks.

In this study, we did not directly address interference effects between sequence learning and a secondary distractor task. For instance, Goschke and Bolte (2012) show in their Experiment 3 that interference is virtually complete if the distractor task is highly similar to sequence features (e.g., phonological distractor task for a letter sequence), but learning can be observed for a secondary sequence that is dissimilar to the distractor task (e.g., phonological distractor task and a sequence of spatial locations). There are two reasons why we think this finding is not problematic for the view presented here. First, the problem that finding above-zero learning can be explained not only by absence of interference, but also incomplete destructive interference, applies to this finding, too. Second, if learning proceeds in a unitary learning system, such a system is inevitably dependent on attention: A learning situation with attention diverted by a distractor task will evidently hamper learning, and interference will likely increase with sequence-distractor similarity—potentially because distractors are involuntarily encoded into memory at the position of the last item of the distractor-similar sequence (c.f., Oberauer, Farrell, Jarrold, Pasiecznik, & Greaves, 2012).

From a theoretical perspective, arguably the most pressing question pertaining the single-system view is what determines the extent of interference between multiple sequences (or a sequence and a distractor task). An obvious candidate moderator is the degree of similarity between sequence features, but such a construct requires further specification (c.f., Guest & Lamberts, 2011; Oberauer & Lin, 2017). Interestingly, this question is analogous to another question that arose in the context of the multiple-systems view: Here, whether or not information is processed in the same or different modules is used to explain interference effects, and the critical question is what constitutes the dimensions along which processing modules are separated. It could be fruitful to get some inspiration from this line of research: Studies by Haider and colleagues (Eberhardt et al., 2017; Haider et al., 2018) indicate that interference between multiple streams of information is not as much a function of modality or whether stimuli or response sequences overlap – instead, they find that abstract features, for instance, spatial vs. non-spatial information, determine whether interference occurs. Haider and colleagues explain these findings by assuming that the modules of the unidimensional learning system are separated along such abstract feature dimensions. From a single-system perspective such as the one presented here, these findings may also be explained by more or less similarity between sequence features: For instance, two spatial sequences might be more similar to each other than a color and a spatial sequence, effectively leading to more interference.

### Summary and outlook

Previous research that aimed at testing the postulated modularization within a unidimensional learning system frequently followed the rationale that the parallel acquisition of multiple sequences is only possible if such modularization prevents (destructive) interference between sequences. However, finding above-zero learning for both sequences is insufficient to conclude that learning proceeded separately in a modularized fashion: If, instead, both sequences were processed jointly in a unitary learning system, and interference between sequences was only incomplete, it is still possible to find such above-zero learning. Here we demonstrated that an instance model of the SRTT that assumes a unitary representation of sequence information is indeed well able to predict the parallel acquisition of multiple uncorrelated sequences.

By contrast, *absence of interference* is a much stronger empirical prediction deduced from the view that implicit learning proceeds in a strictly modularized sequence-learning system. Therefore, an obvious route for future research is to conduct empirical studies that directly assess whether or not learning of individual sequences under dual-sequence conditions is comparable to single-sequence conditions. If such studies were to find that interference is indeed absent under dual-sequence conditions, this would challenge the single-system explanation; it could only be reconciled with such a finding by imposing auxiliary assumptions, for instance, that under dual-task conditions higher task demands bolster attention and, consequently, learning. If, instead, such studies were to find only incomplete interference between sequences, the dual-systems view might be amended by assuming that the modules comprising the unidimensional system are not strictly separated, but are more interrelated (c.f., Seger, 1994), and are therefore also prone to interference between sequences. Eberhardt et al. (2017) already realized conditions under which such absence of interference could be tested. In their Experiment 2, stimuli were presented in locations that followed a sequential structure; for some participants, stimulus colors adhered to another sequence, while for other participants, no additional sequence was present. Descriptively, learning of the stimulus-location sequence was comparable between these two conditions, suggesting that the color sequence indeed did not interfere with the location sequence.

Another possible route is to expand on the study design utilized in Experiment 3 of Goschke and Bolte (2012), where the authors manipulated the similarity of a distractor task with both sequences. There, they found that the extent to which a distractor task interfered with either sequence corresponded to its similarity with the respective sequence. As we argued in our introduction, such a finding does not provide strong evidence for modularization, because a single-sytem view would also predict stronger interference for similar (compared to nonsimilar) distractor tasks. However, such a study design could be analyzed in a state-trace fashion (Dunn & Kirsner, 1988; Stephens, Matzke, & Hayes, 2019) to provide stronger support for modularization. If the learning scores for both sequences followed a non-monotonic function of distractor-task similarity, the single-system view could not explain such a finding with increased task demands or attentional tuning under dual-sequence conditions.

To conclude, parallel acquisition of two uncorrelated sequences does not provide firm evidence for encapsulated processing in a modularized sequence-learning system. There are, however, clear paths for future research to clarify whether or not, or to what extent, implicit sequence learning relies on encapsulated processing modules.

## Data Accessibility Statement

Code and materials necessary to reproduce the analyses reported in this article are available at https://github.com/methexp/cpl-minerva.

## Footnotes

Goschke and Bolte (2012) additionally tested the independence of the learned representation using the bivariate correlation of the individual learning scores, formed as the difference of reaction times for regular and non-regular transitions – recent work (Malejka, Vadillo, Dienes, & Shanks, 2021; Rouder, Kumar, & Haaf, n.d.) shows, however, that correlations of difference scores cannot be reliably estimated under the usual conditions in cognitive psychology (number of participants, number of trials per participant). Therefore, the zero correlation found by Goschke and Bolte (2012) in their experiments cannot be interpreted.

Assuming, in addition, a lack of statistical power for obtaining the weaker learning effects in the uncorrelated sequences.

## References

[1] Aust, F., & Barth, M. (2022). papaja: Prepare reproducible APA journal articles with R Markdown. Retrieved from https://github.com/crsh/papaja

[2] Barth, M. (2022). tinylabels: Lightweight variable labels. Retrieved from https://cran.r-project.org/package=tinylabels

[3] Cleeremans, A., & Jiménez, L. (2002). Implicit learning and consciousness: A graded, dynamic perspective. Implicit Learning and Consciousness (pp. 1–40).

[4] Dunn, J. C., & Kirsner, K. (1988). Discovering functionally independent mental processes: The principle of reversed association. Psychological Review, 95(1), 91–101. DOI: 10.1037//0033-295X.95.1.91 3353477

[5] Eberhardt, K., Esser, S., & Haider, H. (2017). Abstract feature codes: The building blocks of the implicit learning system. Journal of Experimental Psychology: Human Perception and Performance, 43(7), 1275–1290. DOI: 10.1037/xhp0000380 28287760

[6] Frost, R., Armstrong, B. C., Siegelman, N., & Christiansen, M. H. (2015). Domain generality versus modality specificity: The paradox of statistical learning. Trends in Cognitive Sciences, 19(3), 117–125. DOI: 10.1016/j.tics.2014.12.010 25631249 PMC4348214

[7] Goschke, T., & Bolte, A. (2012). On the modularity of implicit sequence learning: Independent acquisition of spatial, symbolic, and manual sequences. Cognitive Psychology, 65(2), 284–320. DOI: 10.1016/j.cogpsych.2012.04.002 22621762

[8] Guest, D., & Lamberts, K. (2011). The time course of similarity effects in visual search. Journal of Experimental Psychology: Human Perception and Performance, 37(6), 1667–1688. DOI: 10.1037/a0025640 22004196

[9] Haider, H., Esser, S., & Eberhardt, K. (2018). Feature codes in implicit sequence learning: Perceived stimulus locations transfer to motor response locations. Psychological Research, 1–12. DOI: 10.1007/s00426-018-0980-0 29340773

[10] Haider, H., Esser, S., & Eberhardt, K. (2020). Feature codes in implicit sequence learning: Perceived stimulus locations transfer to motor response locations. Psychological Research, 84(1), 192–203. DOI: 10.1007/s00426-018-0980-0 29340773

[11] Hintzman, D. L. (1984). MINERVA 2: A simulation model of human memory. Behavior Research Methods, Instruments, & Computers, 16(2), 96–101. DOI: 10.3758/BF03202365

[12] Jamieson, R. K., & Mewhort, D. J. K. (2009). Applying an exemplar model to the serial reaction-time task: Anticipating from experience. The Quarterly Journal of Experimental Psychology, 62(9), 1757–1783. DOI: 10.1080/17470210802557637 19219752

[13] Keele, S. W., Ivry, R., Mayr, U., Hazeltine, E., & Heuer, H. (2003). The cognitive and neural architecture of sequence representation. Psychological Review, 110(2), 316–339. DOI: 10.1037/0033-295X.110.2.316 12747526

[14] Kelly, M. A., Mewhort, D. J. K., & West, R. L. (2017). The memory tesseract: Mathematical equivalence between composite and separate storage memory models. Journal of Mathematical Psychology, 77, 142–155. DOI: 10.1016/j.jmp.2016.10.006

[15] Kemény, F., & Meier, B. (2016). Multimodal sequence learning. Acta Psychologica, 164, 27–33. DOI: 10.1016/j.actpsy.2015.10.009 26708623

[16] Malejka, S., Vadillo, M. A., Dienes, Z., & Shanks, D. R. (2021). Correlation analysis to investigate unconscious mental processes: A critical appraisal and mini-tutorial. Cognition, 212, 104667. DOI: 10.1016/j.cognition.2021.104667 33975175

[17] Mayr, U. (1996). Spatial attention and implicit sequence learning: Evidence for independent learning of spatial and nonspatial sequences. Journal of Experimental Psychology: Learning, Memory & Cognition, 22(2), 350–364. DOI: 10.1037/0278-7393.22.2.350 8901340

[18] Meier, B., & Cock, J. (2010). Are correlated streams of information necessary for implicit sequence learning? Acta Psychologica, 133(1), 17–27. DOI: 10.1016/j.actpsy.2009.08.001 19717137

[19] Meier, B., Weiermann, B., & Cock, J. (2012). Only correlated sequences that are actively processed contribute to implicit sequence learning. Acta Psychologica, 141(1), 86–95. DOI: 10.1016/j.actpsy.2012.06.009 22864311

[20] Navarro, D. J., & Fuss, I. G. (2009). Fast and accurate calculations for first-passage times in Wiener diffusion models. Journal of Mathematical Psychology, 53(4), 222–230. DOI: 10.1016/j.jmp.2009.02.003

[21] Nissen, M. J., & Bullemer, P. (1987). Attentional requirements of learning: Evidence from performance measures. Cognitive Psychology, 19(1), 1–32. DOI: 10.1016/0010-0285(87)90002-8

[22] Oberauer, K., Farrell, S., Jarrold, C., Pasiecznik, K., & Greaves, M. (2012). Interference between maintenance and processing in working memory: The effect of item-distractor similarity in complex span. Journal of Experimental Psychology: Learning, Memory, and Cognition, 38(3), 665–685. DOI: 10.1037/a0026337 22141748

[23] Oberauer, K., & Lin, H.-Y. (2017). An interference model of visual working memory. Psychological Review, 124(1), 21–59. DOI: 10.1037/rev0000044 27869455

[24] R Core Team. (2022). R: A language and environment for statistical computing. Vienna, Austria: R Foundation for Statistical Computing. Retrieved from https://www.R-project.org/

[25] Ratcliff, R. (1978). A theory of memory retrieval. Psychological Review, 85(2), 59–108. DOI: 10.1037/0033-295X.85.2.59

[26] Rouder, J., Kumar, A., & Haaf, J. M. (n.d.). Why most studies of individual differences with inhibition tasks are bound to fail. DOI: 10.31234/osf.io/3cjr5 PMC10728261 37450264

[27] Rowland, L. A., & Shanks, D. R. (2006). Attention modulates the learning of multiple contingencies. Psychonomic Bulletin & Review, 13(4), 643–648. DOI: 10.3758/BF03193975 17201364

[28] Seger, C. A. (1994). Implicit learning. Psychological Bulletin, 115(2), 163–196. DOI: 10.1037/0033-2909.115.2.163 8165269

[29] Shanks, D. R. (2010). Learning: From association to cognition. Annual Review of Psychology, 61(1), 273–301. DOI: 10.1146/annurev.psych.093008.100519 19575617

[30] Shanks, D. R., & St. John, M. F. (1994). Characteristics of dissociable human learning systems. Behavioral and Brain Sciences, 17(3), 367–395. DOI: 10.1017/S0140525X00035032

[31] Shimamura, A. P., & Wickens, T. D. (2009). Superadditive memory strength for item and source recognition: The role of hierarchical relational binding in the medial temporal lobe. Psychological Review, 116(1), 1–19. DOI: 10.1037/a0014500 19159146

[32] Singmann, H., Brown, S., Gretton, M., & Heathcote, A. (2022). rtdists: Response time distributions. Retrieved from https://CRAN.R-project.org/package=rtdists

[33] Stephens, R. G., Matzke, D., & Hayes, B. K. (2019). Disappearing dissociations in experimental psychology: Using state-trace analysis to test for multiple processes. Journal of Mathematical Psychology, 90, 3–22. DOI: 10.1016/j.jmp.2018.11.003

[34] Timmermans, B., & Cleeremans, A. (2015). How can we measure awareness? An overview of current methods. In M. Overgaard (Ed.), Behavioral Methods in Consciousness Research (pp. 21–46). Oxford University Press. DOI: 10.1093/acprof:oso/9780199688890.003.0003

[35] Voss, A., Nagler, M., & Lerche, V. (2013). Diffusion models in experimental psychology. Experimental Psychology, 60(6), 385–402. DOI: 10.1027/1618-3169/a000218 23895923

[36] Wagenmakers, E.-J. (2009). Methodological and empirical developments for the Ratcliff diffusion model of response times and accuracy. European Journal of Cognitive Psychology, 21(5), 641–671. DOI: 10.1080/09541440802205067

